# Comparison of humoral neuroinflammation and adhesion molecule expression in two models of experimental intracerebral hemorrhage

**DOI:** 10.1186/2040-7378-3-11

**Published:** 2011-10-03

**Authors:** Arthur Liesz, Moritz Middelhoff, Wei Zhou, Simone Karcher, Sergio Illanes, Roland Veltkamp

**Affiliations:** 1 Department of Neurology, University Heidelberg, Im Neuenheimer Feld 400, 69120 Heidelberg, Germany; 2 UTAC, Hospital Clínico Universidad de Chile, Santos Dumont 999, Santiago, Chile

## Abstract

**Background:**

Inflammatory cascades contribute to secondary injury after intracerebral hemorrhage (ICH) via humoral factors and cell-mediated cytotoxicity. Several experimental models were previously developed to analyze post-hemorrhagic neuroinflammation. However, neuroinflammatory markers have not been compared face-to-face between these models so far, and therefore, pathophysiological conclusions drawn from only one individual model may not be valid.

**Methods:**

We compared neuroinflammatory pathways in the two most common murine models: striatal injection of autologous blood or collagenase. Expression of pro- and anti-inflammatory cytokines (IL-1, TNF-α, IFN-γ, IL-6, TGF-β and IL-10) as well adhesion molecule expression (VCAM-1, ICAM-1) was analyzed by RT-PCR at several time points after ICH induction. Outcome and physiological parameters were compared between the models.

**Results:**

Both models induced a profound and dynamic increase in the expression of pro-inflammatory cytokines and adhesion molecules. However, blood injection resulted in significantly more pronounced alteration of these markers than collagenase injection. This difference was associated with worse outcome after blood injection compared to the collagenase model despite equal ICH volumes.

**Conclusions:**

This is the first study performing a face-to-face comparison of neuroinflammatory pathways in the two most widely used murine ICH models, revealing substantial differences between the models. This discrepancies need to be taken into account in designing future studies employing experimental ICH models, especially when analyzing neuroinflammatory pathways and therapies.

## Background

Intracerebral hemorrhage (ICH) represents about 10-15% of all strokes. Compared to ischemic stroke, it is associated with substantially higher morbidity and mortality [1,2]. In contrast to the major importance of ICH as a health care problem, no specific therapy for ICH is available until now. Moreover, the current understanding of crucial pathophysiological mechanisms is limited [3].

To improve the search for potential therapeutic targets in ICH, several rodent models of ICH have been developed [4-6]. The most widely used techniques are direct injection of either blood or of collagenase, respectively [4,7,8]. Collagenase digests the extracellular matrix of small vessels and dose-dependently causes blood extravasation. Although both methods fail to reflect important components of the pathophysiology of ICH in patients [9], they allow to study the impact of extravascular blood on the brain parenchyma. However, differences in signaling cascades of pathophysiologically relevant mechanisms between these models are likely.

Secondary inflammatory damage to brain tissue has emerged as a common link between differing types of central nervous system disorders [3,10-12]. Several studies using rodent ICH models have suggested a significant contribution of inflammatory pathways to the pathophysiology of ICH [13,14]. Inflammatory mechanisms have been examined in both the blood and the collagenase injection model [13]. The main inflammatory pathways after ICH are leukocyte migration into the brain, microglial activation, cerebral cytokine expression and production of other neurotoxic factors including matrix metalloproteinases and reactive oxygen species [14]. Unfortunately, specific aspects of neuroinflammation have not been compared face-to-face between these models so far, and therefore, pathophysiological conclusions drawn from only one individual model may not be valid.

The aim of the present study therefore was to compare the dynamic changes in cerebral expression of important cytokines and adhesion molecules as key markers of neuroinflammation in the autologous blood injection and the collagenase injection model of intracerebral hemorrhage.

## Materials and methods

### Animals

In accordance with national guidelines for the use of experimental animals the study was conducted using age-matched, mature male mice (C57BL/6, Charles River Laboratories, 10-12 weeks of age, body weight 20-25 g). The protocols were approved by the governmental committees (Regierungspraesidium Karlsruhe, Germany).

### Animal experiments

Spontaneously breathing mice were anesthetized with halothane (1%-1.5%) in an oxygen/air mixture. After placing the animals in a stereotactic frame (Stoelting, USA) with a mouse adaptor (Model 51625, Stoelting, USA) a borehole was drilled at 2 mm left and 0.5 mm anterior to the bregma as previously described [15,16].

Using an infusion system containing a 25 G needle connected (1) to a 50 μl microsyringe (Hamilton, Switzerland) for the injection of 30 μL autologous blood (taken from the retro-orbital plexus) or (2) to a 10 μL microsyringe (SGE, Austria) for the injection of 0.5 μL sterile saline containing 0.045 U collagenase type VII (Sigma, Germany) was inserted into the striatum at 3.5 mm depth from scull. The duration of both operations of approximately 45 min was matched to avoid an interference caused by perioperative management including anesthesia. Sham operation was conducted by the same procedure and with the same duration as for ICH induction including placement of the microneedle but without injection of fluid.

### Hematoma measurement

In separate experiments, mice were euthanized 24 h after ICH induction (n = 7 per group). 40 μm thick coronal cryosections were cut every 400 μm. Unstained sections were scanned at 600 dpi. The hemorrhagic area was marked and measured using a public domain image analysis program (ImageJ). Total hematoma volume was obtained by integrating measured areas and distance between sections.

### RNA isolation and real-time polymerase chain reaction

RNA was isolated from separated cerebral hemispheres using the RNeasy kit (Qiagen). Reverse transcription was performed with the High Capacity complementary DNA Archive Kit and real-time polymerase chain reaction with SYBR-Green assays (on an ABI7500 real-time polymerase chain reaction system (Applied Biosystems). Primers were purchased as ready-to-use primer sets for each gene (all from Super Array, IL-1β: PPM03109E, IL-10: PPM03017B, IL-6: PPM03015A, TNF-α: PPM03113A, IFN-γ: PPM03121A, TGF-β: PPM02991A, VCAM-1: PPM03208B, ICAM-1: PPM03196A). All assays were run in duplicates. A linear dilution-amplification curve was obtained from diluted pooled samples. Using this curve the expression of each gene was quantified relative to the expression of the housekeeping gene encoding for peptidylprolyl isomerase A (cyclophilin), according to the relative standard curve method. Analysis of mRNA expression was performed blinded to treatment groups.

### Statistical analysis

All values in bar graphs are expressed as mean ± standard deviation (SD). We used the Mann-Whitney U-Test for comparison of two groups. For three or more groups we applied the Kruskal-Wallis H test with Dunn's post hoc testing, using GraphPad Prism 5 software. P < 0.05 was considered statistically significant.

## Results

### Differing outcome despite same ICH volume in the autologous blood and colleganase injection model

**Figure 1:**
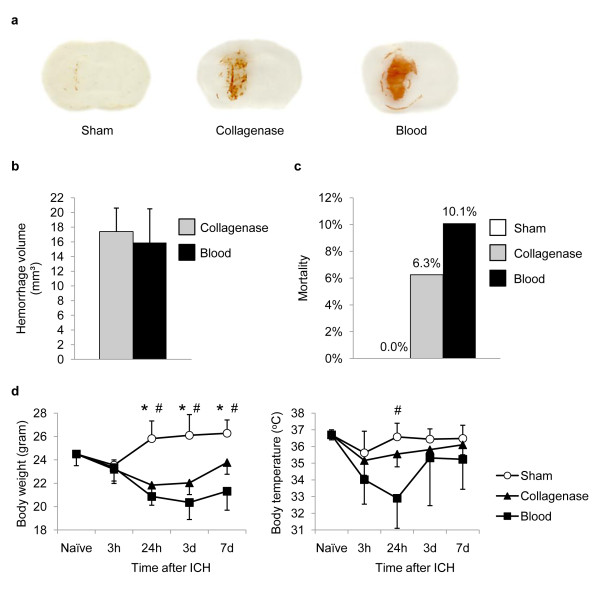
**Characterization of the used ICH models**. (a) Representative images from the same anatomical region at 24 h after sham operation, intrastriatal injection of 0.045 U collagenase ("Collagenase") or 30 μl autologous blood ("Blood") as used for measurement of hematoma volume. (b) Hemorrhage volume was determined 24 h after ICH by integration of serial coronal sections, no difference between the collagenase and blood injection model was detected (n = 7, 2 individual experiments). (c) Overall mortality was determined for all animals used in this study treated by sham operation or ICH induction by one of both models, respectively. (d) Body weight and rectal body temperature was measured in naïve mice and at the indicated time points after sham operation or ICH induction by the respective model (n = 6-11 per group).* p < 0.05 between Sham and Collagenase; # p < 0.05 between Sham and Blood.

Both models were optimized to induce ICH of comparable blood volume as used in most previous experimental ICH studies [15-17]. Injection of either 30 μL autologous blood or 0.045 U collagenase VII into the left striatum resulted in an intracerebral hematoma volume of approx. 15-17 mm^3 ^(Figure 1a, b). However, 24 h after ICH induction the hemorrhagic area appeared generally denser in the blood injection model than after collagenase-induced ICH (Figure 1a). Total mortality of mice differed between the experimental models in this study (Figure 1c). While no mice died in the sham group, overall mortality was 6.3% in the collagenase injection model (3/48 operated mice) and 10.1% in the autologous blood injection model (8/79 operated mice). Both ICH models were associated with a pronounced weight loss compared to sham operated mice (Figure 1d). However, only mice undergoing blood-injection ICH showed a significant but transient hypothermia 24 h after ICH compared to the sham group (Figure 1d).

### Cerebral expression patterns of pro-inflammatory cytokines

**Figure 2:**
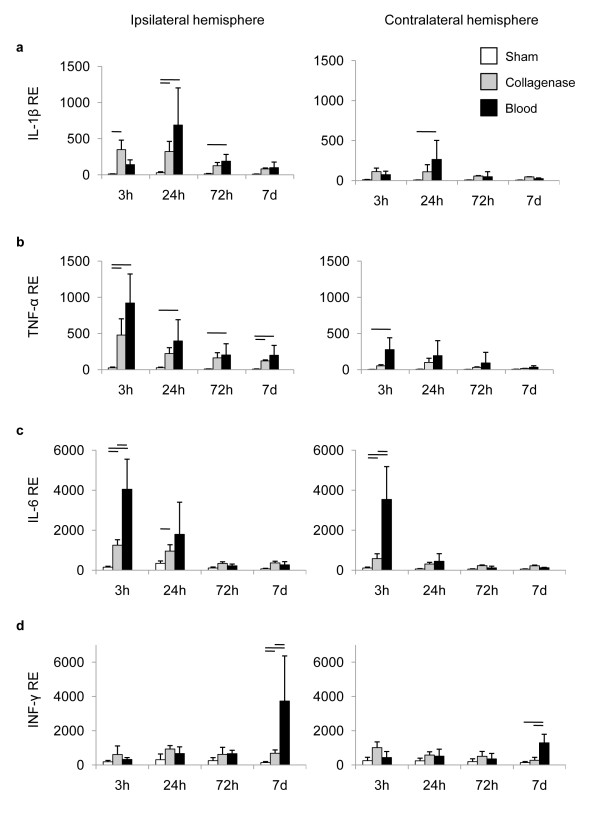
**Expression patterns of pro-inflammatory cytokines after ICH**. Relative expression (RE) was determined for (a) IL-1β, (b) TNF-α, (c) IL-6 and (d) IFN-γ at the indicated time points in the hemorrhagic (ipsilateral) and non-hemorrhagic (contralateral) hemispheres after sham operation or ICH induction by the collagenase or autologous blood injection model. Horizontal lines indicate significant statistical differences (p < 0.05) between the indicated groups (n = 5 per group, 2-3 individual experiments).

We analyzed the relative expression of key pro-inflammatory cytokines at 3 h, 24 h, 72 h and 7d after ICH induction in both ICH models and after sham operation (Figure 2). IL-1β and TNF-α have been well characterized as the main neurotoxic cytokines of microglial source after ischemic and traumatic brain injury [18-20]. We detected a significant increase of both cytokines in the hemorrhagic hemispheres of mice undergoing ICH induction in both models compared to the sham group (Figure 2a, b). Increased cytokine expression occurred very early after ICH and peaked already at 3 h for TNF-α and at 24 h for IL-1β. Autologous blood injection, but not collagenase injection, induced a significant increase in IL-1β at 24 h and TNF-α expression 3 h after ICH in the contralateral, non-hemorrhagic hemisphere (Figure 2a, b). IL-6 is a pleiotropic cytokine produced by various cellular sources in the brain after CNS lesions [21]. IL-6 expression was significantly increased 6 h after ICH in both ICH models and at 24 h in the collagenase model compared to sham (Figure 2c). Interestingly, autologous blood injection resulted in significantly higher IL-6 expression than collagenase injection in the hemorrhagic and the non-hemorrhagic hemisphere at 6 h (Figure 2c). Interferon-gamma (IFN-γ) was analyzed as the key neurotoxic cytokine produced by activated lymphocytes [22,23]. IFN-γ expression did not increase within the first 3d after ICH, but was significantly overexpressed 7d after ICH in both ICH models compared to sham (Figure 2d). Blood injection induced a significantly higher, approx. 5-fold IFN-γ expression compared to the collagenase-injection model and also increased IFN-γ expression in the contralateral hemisphere (Figure 2d).

### Cerebral expression patterns of anti-inflammatory cytokines

**Figure 3:**
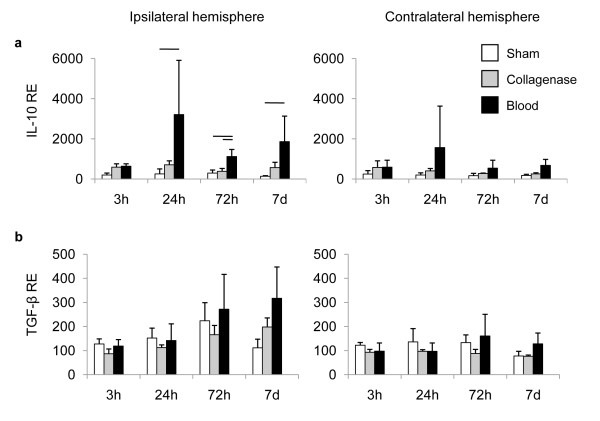
**Expression patterns of anti-inflammatory cytokines after ICH**. We determined the relative expression (RE) of (a) IL-10 and (b) TGF-β mRNA at the indicated time points in the hemorrhagic (ipsilateral) and non-hemorrhagic (contralateral) hemispheres after sham operation or ICH induction by the collagenase or autologous blood injection model. Horizontal lines indicate significant statistical differences (p < 0.05) between the indicated groups (n = 5 per group, 2-3 individual experiments).

Previous reports from our and other groups have shown a neuroprotective effect of the anti-inflammatory cytokines IL-10 and TGF-β, respectively, after CNS lesions [20,24-26]. We detected a significant increase of IL-10 mRNA expression only in the autologous blood injection model (Figure 3a). An up to 3-fold increase in IL-10 expression occurred in the hemorrhagic hemispheres 24 h to 7d after striatal blood injection, while collagenase-induced ICH did not affect IL-10 expression (Figure 3a). In contrast, TGF-β transcription was not altered by any experimental protocol at any time point after ICH induction or sham operation (Figure 3b).

### Expression of cerebral adhesion molecules after experimental ICH

**Figure 4:**
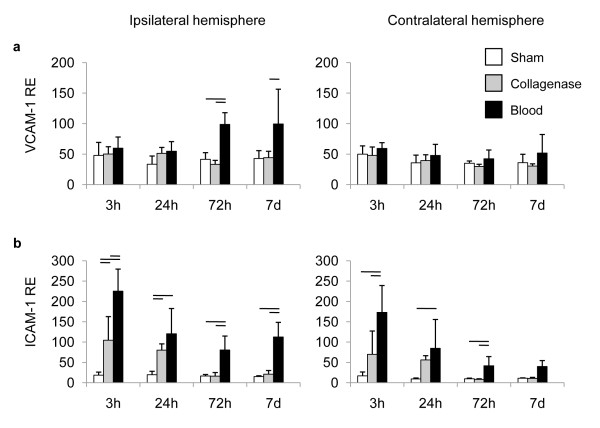
**Expression of cerebral endothelial adhesion molecules after ICH**. (a) VCAM-1 and (b) ICAM-1 expression was measured at the indicated time points in the hemorrhagic (ipsilateral) and non-hemorrhagic (contralateral) hemispheres after sham operation or ICH induction by the collagenase or autologous blood injection model. Horizontal lines indicate significant statistical differences (p < 0.05) between the indicated groups (n = 5 per group, 2-3 individual experiments).

The endothelial adhesion molecules VCAM-1 and ICAM-1 have been associated with transendothelial diapedesis of leukocytes and subsequent secondary neuroinflammation in various non-inflammatory CNS disorders and particularly experimental ischemic stroke [27-29]. Significant VCAM-1 upregulation was observed only in the hemorrhagic hemispheres in the autologous blood injection model 3d and 7d after ICH compared to sham and collagenase injection (Figure 4a). Surprisingly, collagenase injection did not significantly alter VCAM-1 expression at any measured time point after ICH induction (Figure 4a). In contrast, ICAM-1 expression was already substantially increased 3 h after ICH induction in both models compared to sham control (Figure 4b). ICAM-1 expression was approx. 12-fold higher than in sham treated animals already 3 h after ICH in the blood injection model and was also significantly higher than in the collagenase group (Figure 4b). A similar pattern, but less pronounced, was observed 24 h after ICH. In contrast, at 3d and 7d after hemorrhage ICAM-1 expression was already normalized again in the collagenase injection model while it remained significantly upregulated in animals with blood-injection ICH.

## Discussion

To the best of our knowledge, this is the first study performing a face-to-face comparison of neuroinflammatory pathways in the two most widely used models of murine experimental intracerebral hemorrhage. The major findings of this work are that (1) experimental ICH induces a profound induction of cerebral cytokine expression, (2) ICH increases the expression of cerebral adhesion molecules, (3) autologous blood injection alters these mechanisms generally more pronounced than collagenase injection, (4) blood injection, but not collagenase, induces neuroinflammatory pathways also in the contralateral hemispheres, and (5) the difference in the extent of ICH-induced neuroinflammation between the 2 models is associated with worse outcome in the blood than the collagenase injection model despite equal hematoma volumes.

The kinetics of cytokine expression within the first week after experimental ICH was quite dynamic. The expression pattern differed distinctively between the analyzed cytokines and showed an early peak for IL-1, TNF-α and IL-6 while IFN-γ was significantly increased only at 7d after ICH. The kinetics of cerebral cytokine expression observed in this study resembled the pattern observed after focal cerebral ischemia [30,31]. There, microglial cells have been identified as the main producers of the early increase in intracerebral IL-1 and TNF-α, while the delayed expression of IFN-γ correlated with the later invasion of lymphocytes-the main source of IFN-γ-to the brain [20,32-34]. VCAM-1 mRNA expression also showed a delayed increase in parallel to IFN-γ expression. This is especially notable because VCAM-1 has previously been shown to be the key adhesion molecule controlling invasion of lymphocytes to brain lesions after ischemic stroke [23,28] and might thereby be of relevance in the cascade of lymphocyte invasion and cerebral IFN-γ production after ICH.

Previous studies have investigated microglia/macrophage activation as well as brain leukocyte invasion after experimental ICH [34-36]. Neutrophils have been shown as the earliest subpopulation invading the brain after ICH in preclinical studies [37]. Moreover, lymphocytes migrated to the lesion at approx. 48-72 h after ICH [35]. A report by Loftspring et al. using FACS analyses of brain homogenates after blood injection has documented an early influx of granulocytes and a delayed invasion of CD4+ and CD8+ T cells [34]. Xue and Del Bigio [36] compared the cellular immune reactions in 3 ICH rodent models, including autologous blood injection and collagenase injection. Interestingly, this study detected distinct differences in the kinetics and magnitude of neutrophile and CD8+ lymphocyte brain influx and microglial activation between the blood and the collagenase injection model in rats as blood injection resulted in earlier microglial activation and CD8+ T cell migration to the brain compared to collagenase-induced ICH.

We detected in this study profound differences in neuroinflammatory pathways between the two most common murine ICH models independent of ICH volume. In general, the autologous blood injection model induced more pronounced alterations in the analyzed immunological pathways in the hemorrhagic hemisphere-pro-inflammatory cytokines, anti-inflammatory cytokines and adhesion molecule expression-compared to the collagenase model. Additionally, only ICH induction by the autologous blood injection model (except for IL-6 expression at 3 h) induced significantly the expression of cytokines and adhesion molecules also in the contralateral, non-hemorrhagic hemisphere. Previous reports analyzing intracerebral cytokine expression in murine ICH models have used either autologous blood injection [38,39] or collagenase-induced ICH [40,41]. There, both models resulted in increased pro- and anti-inflammatory cytokine expression. However, the present study shows substantial differences in cytokine expression between ICH models.

Previous reports have documented an increase in cerebral ICAM-1 expression after ICH in the blood injection model [39,42,43]. We could reproduce these findings and detected additionally ICAM-1 induction in the collagenase model which was weaker compared to blood injection. VCAM-1 has been proposed as a therapeutic target in ischemic stroke and traumatic brain injury [23,44]. This is the first study to characterize VCAM-1 expression after ICH. Surprisingly, we observed a significant increase in VCAM-1 expression in the blood injection but not the collagenase injection model. The relevance of this major discrepancy between the two ICH models on leukocyte invasion, neuroinflammation and outcome needs to be further investigated because of the key role of VCAM-1 in the inflammatory cascade after brain injury [28].

Developing new therapies for ICH is the ultimate goal of translational stroke research. So far more than 30 studies have investigated anti-inflammatory therapies targeting various cells and molecules in animal models of ICH [14,45]. Experiments in these studies were performed in different species, used different methods to induce ICH, and produced different hematoma volumes in relation to the volume of the affected hemisphere. As long as the inflammatory cascades participating in the pathophysiology of human ICH are largely unknown, translational experiments have to take potential discrepancies of experimental models into account and should test hypotheses in different models.

## Conflict of interest

The authors declare that they have no competing interests.

## Authors' contributions

AL designed and performed experiments, analyzed data, and wrote the manuscript; MM, WZ, SK and SI. performed experiments and analyzed data; RV. initiated and directed the entire study, designed experiments, analyzed data, and wrote the manuscript.

All authors have read and approved the final version of the manuscript.
